# Pulmonary fibrosis as the sole manifestation of anti-Ku antibody positivity in the absence of myositis: A case report

**DOI:** 10.1016/j.rmcr.2025.102165

**Published:** 2025-01-02

**Authors:** Angelo Nigro

**Affiliations:** Department of Rheumatology of Lucania - UOSD of Rheumatology, "Madonna delle Grazie" Hospital, Matera, Italy

**Keywords:** Anti-Ku antibodies, Pulmonary fibrosis, Myositis, Interstitial lung disease, Autoimmunity, Case report

## Abstract

**Background:**

Anti-Ku antibodies are autoantibodies directed against the Ku protein complex involved in DNA repair. They are typically associated with overlap syndromes featuring polymyositis and systemic sclerosis. Isolated pulmonary involvement without myositis is exceedingly rare.

**Case presentation:**

We report the case of a 70-year-old male, a former smoker with an 18-year smoking history who quit 20 years ago, presenting with a one-year history of progressive dyspnea and dry cough. High-resolution computed tomography (HRCT) revealed pulmonary fibrosis with areas of ground-glass opacities. Laboratory tests showed antinuclear antibodies at a titer of 1:2560 with a speckled pattern and positivity for anti-Ku antibodies. Creatine phosphokinase levels were within normal limits. There were no clinical signs of myositis, myalgia, skin manifestations, or Raynaud's phenomenon.

**Conclusion:**

This case underscores the rarity of pulmonary fibrosis as the sole clinical manifestation associated with anti-Ku antibody positivity in the absence of myositis. Clinicians should consider testing for anti-Ku antibodies in patients with idiopathic interstitial lung disease, even when muscular and cutaneous symptoms are absent.

## Highlights

• **Rare Case Presentation**: This case demonstrates the unusual occurrence of pulmonary fibrosis as the sole clinical manifestation of anti-Ku antibody positivity, in the absence of myositis or other systemic features.
• **Clinical Implication**: It emphasizes the importance of comprehensive autoimmune testing, including anti-Ku antibodies, in patients presenting with idiopathic interstitial lung disease without muscular or cutaneous symptoms.
• **TreatmentOutcome**: The patient responded to corticosteroid therapy, highlighting the potential benefit of early immunosuppressive treatment in managing similar cases.

## 1 Introduction

Anti-Ku antibodies are autoantibodies directed against the Ku heterodimer protein complex, consisting of 70 kDa (Ku70) and 80 kDa (Ku80) subunits, which play a crucial role in DNA repair and transcription regulation. These antibodies are most commonly associated with overlap syndromes involving polymyositis and systemic sclerosis but are rare overall, with a prevalence of less than 1 % in connective tissue diseases [1]. Pulmonary involvement is common in anti-Ku-positive patients but usually occurs alongside muscular and cutaneous manifestations [2]. We present a rare case where pulmonary fibrosis was the sole clinical manifestation associated with anti-Ku antibody positivity, in the absence of myositis or other systemic features.

## 2 Case Presentation

A 70-year-old Caucasian male presented with a one-year history of progressive exertional dyspnea and persistent dry cough. He was a former smoker with an 18-pack-year smoking history, having ceased smoking 20 years prior to presentation. He had no significant past medical history. The patient resided in an urban environment and reported no exposure to rural settings, birds, or mold that could potentially contribute to interstitial lung disease. Furthermore, he had no documented history of occupational exposure to metals, chemicals, or other agents known to be associated with pulmonary conditions. Physical examination revealed bilateral basal crackles on lung auscultation. There were no skin rashes, muscle weakness, joint swelling, digital ulcers, or signs suggestive of Raynaud's phenomenon. Vital signs were within normal limits.

Laboratory investigations revealed mildly elevated erythrocyte sedimentation rate and C-reactive protein levels. Creatine phosphokinase (CPK) levels were normal (95 U/L; reference range: 30–200 U/L). Antinuclear antibodies (ANA) were positive at a titer of 1:2560 with a speckled pattern. An extractable nuclear antigen (ENA) panel showed positivity for anti-Ku antibodies (ELISA), while other antibodies including anti-Scl-70, anti-RNP, anti-Jo-1, and anti-Sm were negative. Rheumatoid factor and anti-cyclic citrullinated peptide antibodies were also negative. Tests for anti-PL7, anti-PL12, anti-OJ, and anti-EJ were not performed and are listed as a limitation of this case.

**Fig. 1:**
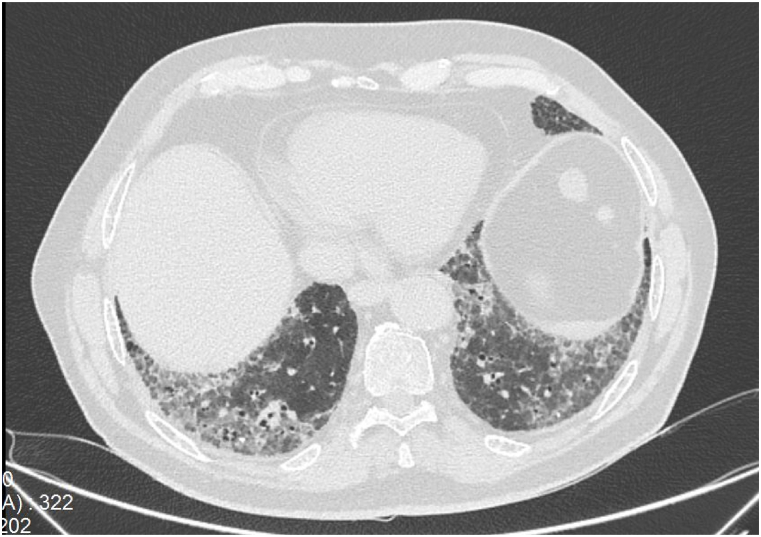
High-resolution computed tomography (HRCT) scan of the chest at the level of the lower lung fields, showing signs of pulmonary fibrosis, including bilateral interstitial infiltrates with reticular patterns and areas of ground-glass opacities.

High-resolution computed tomography (HRCT) of the chest demonstrated bilateral interstitial infiltrates with reticular patterns and areas of ground-glass opacities, predominantly in the lower lobes, consistent with pulmonary fibrosis (Fig. 1). Pulmonary function tests showed a forced vital capacity (FVC) of 65 % of predicted, a total lung capacity (TLC) of 70 % of predicted, forced expiratory volume in 1 s (FEV₁) at 70 % of predicted, a FEV₁/FVC ratio of 0.8, and a diffusing capacity for carbon monoxide (DLCO) at 55 % of predicted.

Given the absence of clinical signs of myositis, electromyography and muscle biopsy were deemed unnecessary. The pulmonologists evaluated the option of performing both bronchoscopy and lung biopsy; however, the patient declined to proceed with these diagnostic interventions.

The diagnosis was interstitial lung disease associated with anti-Ku antibody positivity, without evidence of myositis or other systemic autoimmune features.

The patient was started on corticosteroid therapy (prednisone 0.5 mg/kg/day) and monitored for response and potential side effects. Follow-up imaging at six months showed no changes compared to the initial CT scan, but the patient reported significant clinical improvement.

## 3 Discussion

Anti-Ku antibodies are rare and primarily associated with overlap syndromes combining features of polymyositis and systemic sclerosis [1,3]. The Ku antigen is involved in non-homologous end joining during DNA repair [4]. While pulmonary involvement is common in patients with anti-Ku antibodies, it typically occurs alongside muscular and cutaneous manifestations [2,5].

Casal-Dominguez et al. [2] conducted a study on the phenotype of myositis patients with anti-Ku autoantibodies and found that these patients often present with interstitial lung disease in addition to myositis. However, cases where pulmonary fibrosis is the sole manifestation are exceedingly rare. Our case is unique as the patient presented solely with pulmonary fibrosis, without any clinical or laboratory evidence of myositis, skin involvement, or other systemic features. Normal CPK levels and the absence of muscle weakness or myalgia ruled out myositis.

The patient's smoking history, although significant, may not fully explain the extent and pattern of pulmonary fibrosis observed. Smoking is a known risk factor for interstitial lung disease; however, the presence of high-titer ANA and anti-Ku antibodies suggests an autoimmune etiology.

The pathogenesis of pulmonary fibrosis in anti-Ku-positive patients without myositis remains speculative. It is hypothesized that anti-Ku antibodies may directly contribute to pulmonary fibrosis by targeting the Ku proteins expressed in pulmonary tissues, leading to immune-mediated damage [6]. Alternatively, the antibodies might serve as markers for an underlying autoimmune process confined to the lungs.

Although lung biopsy was not performed in this case, the recent multicenter study [7] demonstrates the utility of cryobiopsy for diagnosing ILD secondary to connective tissue diseases. However, the pneumologists did not consider this necessary based on the presented findings.

Treatment guidelines for anti-Ku antibody-associated interstitial lung disease are not well established due to the rarity of such cases. Immunosuppressive therapy with corticosteroids, alone or in combination with other agents like mycophenolate mofetil or rituximab, has been used with variable success [8,9]. In our patient, corticosteroid therapy stabilized the disease, highlighting the potential benefit of early immunosuppression.

This case emphasizes the importance of considering anti-Ku antibody testing in patients with idiopathic interstitial lung disease, even in the absence of muscular or cutaneous symptoms. Early diagnosis and appropriate management may improve clinical outcomes and prevent disease progression.

## 4 Conclusion

Pulmonary fibrosis can be the sole manifestation of anti-Ku antibody positivity in the absence of myositis and other systemic features. Clinicians should be aware of this rare presentation and consider comprehensive autoimmune testing in patients with unexplained interstitial lung disease. Early recognition and treatment are crucial for disease management and improving patient prognosis. It is important to note that additional serological tests, such as anti-PL7, anti-PL12, anti-OJ, and anti-EJ, were not performed in this case, which represents a limitation in the differential diagnosis process. Additionally, the absence of a lung biopsy and certain serological analyses, which could have provided more definitive diagnostic insights, represents a significant limitation of this case.

## Patient consent

Written informed consent was obtained from the patient for the publication of this case report and any accompanying images.

## Funding

No funding was received for this study.

## Declaration of competing interest

The authors declare that they have no known competing financial interests or personal relationships that could have appeared to influence the work reported in this paper.
